# On the Prospective Application of Behavioral Momentum Theory and Resurgence as Choice in the Treatment of Problem Behavior: A Brief Review

**DOI:** 10.3390/bs15050688

**Published:** 2025-05-16

**Authors:** Michael P. Kranak, John Michael Falligant, Chloe Jones, Meredith Stephens, Megan Wessel

**Affiliations:** 1 Department of Human Development and Child Studies, Oakland University, Rochester, MI 48309, USA; 2 Oakland University Center for Autism, Rochester, MI 48309, USA; 3 Department of Psychological Sciences, Auburn University, Auburn, AL 36849, USA

**Keywords:** Behavioral Momentum Theory, problem behavior, relapse, research-to-practice, Resurgence as Choice

## Abstract

Behavioral Momentum Theory (BMT) and Resurgence as Choice (RaC) are two theoretical and quantitative models of behavior that, when applied prospectively, might improve behavioral treatments and increase the likelihood of long-term success. Despite the plausible benefit of using BMT and RaC to guide clinical decision-making, it is unclear how frequently these models are prospectively used in practice. We briefly review contemporary research on BMT and RaC as related to the treatment of problem behavior. We discuss potential barriers and solutions to their prospective application, as well as areas for future research.

## 1. Introduction

Behavioral treatments (e.g., differential reinforcement) are effective in reducing problem behavior (e.g., Greer et al., 2016b). Often, these interventions are initiated by highly trained staff in controlled settings with near-perfect integrity (see Briggs & Greer, 2021). After achieving clinically significant reductions in problem behavior, treatments are then implemented in less-controlled, natural environments (e.g., homes and schools). It is inevitable that in those settings, treatments will be challenged in some way (e.g., treatment integrity errors; Wacker et al., 2011). Unfortunately, when challenges occur, relapse of problem behavior can lead to treatment failure (e.g., Falligant et al., 2021; Kranak & Falligant, 2021; Mitteer et al., 2022; Muething et al., 2020). Put simply, behavior change is fragile, and treatment gains may not be durable enough to withstand common challenges.

Behavioral Momentum Theory (BMT) and Resurgence as Choice (RaC) are two theoretical and quantitative models of behavior that may improve the durability of behavioral treatments (see Greer & Shahan, 2019; see also Smith & Greer, 2023). Durability refers to the extent to which desirable behavior persists, and problem behavior remains abated in the face of challenges (Wacker et al., 2011). These models enable clinicians and researchers to make predictions about future behavior or treatment success and durability based on variables such as reinforcement history for a given response, exposure to extinction, rate and magnitude of reinforcement, and many other factors (Shahan et al., 2020; Shahan & Craig, 2017).

BMT was derived from basic research on schedules of reinforcement (Nevin & Grace, 2000). Analogous to classical physics, BMT predicts that responses with greater momentum will be more resistant to disruption, akin to objects with greater inertia. Just as a massive object in motion is harder to stop, behaviors associated with richer reinforcement contexts are more persistent when challenged. BMT describes the Pavlovian association between contextual stimuli, the reinforcers delivered and reinforcement rates occurring in their presence, and how the pairing of reinforcers/reinforcement rates and contextual stimuli affects response strength (for a detailed overview, see (Nevin et al., 2017); see (Greer et al., 2016a) for a tutorial). RaC proposes that resurgence is governed by the same behavioral processes that underlie choice behavior (see (Greer & Shahan, 2019) for a comprehensive overview and (Laureano & Falligant, 2023) for a tutorial). RaC posits that allocation between target and alternative responding (e.g., aggression and communication responses, respectively) varies according to the relative value of each response alternative over time. This relation is formalized in a derivation of the concatenated matching law and can further be modified using a version of the quantitative law of effect to generate predictions about the absolute rates of target and alternative behavior.

Recent translational and applied research suggests that treatments grounded in these models may yield more durable outcomes than those developed without such frameworks (see Fisher et al., 2022; e.g., Fisher et al., 2018; Saini et al., 2017). For example, Fisher et al. (2018) demonstrated that functional communication training (FCT) with treatment parameters derived from BMT using baseline and treatment rates of reinforcement (i.e., Equation (1) from (Nevin & Shahan, 2011)) decreased rates of problem behavior by approximately two-thirds for all participants and resulted in less relapse when alternative behavior contacted extinction compared to FCT based on standard clinical protocols. Their results support the notion that treatments prospectively designed and based on quantitative models of behavioral persistence may be more successful and durable than those that are not.

Despite the apparent benefits of incorporating these models into the design of treatments for problem behavior, their prospective use remains unclear. These models were largely developed through retrospective applications, analyzing obtained data to quantify functional relations between environmental variables and behavior (Nevin, 2002; Nevin & Shahan, 2011; Shahan & Craig, 2017). Most published demonstrations continue to use this approach, relying on basic or translational preparations, simulated data rather than clinical applications (e.g., Saini et al., 2017), or focusing on areas outside of problem behavior reduction (see Trump et al., 2021). While these retrospective applications are invaluable for understanding behavioral processes, recent research suggests that their greatest potential lies in their prospective use—informing treatment selection and parameterization to enhance long-term durability and mitigate relapse. If we do not leverage these models prospectively, we may be overlooking critical opportunities to optimize treatment outcomes. Clarifying the frequency and conditions under which these models are applied prospectively—particularly in the treatment of problem behavior—may help identify key variables that influence treatment success and reveal barriers to their broader implementation, several of which will be discussed later. We surveyed recently published articles across 10 behavior-analytic journals to ascertain how often BMT and RaC are applied prospectively. We discuss barriers to their prospective application, ways to ameliorate those barriers, and directions for future research.

## 2. Methods: Prevalence of Prospective Applications of BMT and RaC

We hand-searched and reviewed every article published between 2014 and 2024, including advanced online publications, in 10 behavior-analytic journals containing articles on *both* the treatment of problem behavior and BMT or RaC. Hand searches are precedent and commonly used in scoping reviews, or reviews using procedures comparable to scoping reviews, as they allow for thorough foraging through targeted sources. Hand searches involve manually reviewing the contents of selected journals—typically by scanning titles, abstracts, and keywords of each article—rather than using electronic databases. Reviewed journals included the *Journal of Applied Behavior Analysis* (*JABA*), *Journal of the Experimental Analysis of Behavior* (*JEAB*), *Behavior Analysis in Practice (BAP*), *Behavior Analysis: Research and Practice*, *The Psychological Record*, *Behavior Modification*, *Behavioral Interventions*, *Learning and Motivation*, *Behavioural Processes* (*BProc*), and the *European Journal of Behavior Analysis*. We conducted a brief review using comparable methods to a scoping review of these journals as (1) we were interested in the contemporary prospective application of BMT and RaC, (2) no systematic review has been completed on the prospective application of BMT or RaC, and (3) these journals all have a history of publishing on problem behavior and/or BMT and RaC. Thus, our review procedures were appropriate for this topic (see Munn et al., 2018; e.g., Curiel et al., 2023; Hustyi et al., 2023; Kaur et al., 2025).

First, we reviewed every article’s title, keywords, and abstract. If BMT or RaC was mentioned in any one of those three sources, we included the article in the next stage of the review^1^. Second, for any articles identified in the first step, we conducted an ancestral search (i.e., forward/backward search) to identify any potentially relevant articles. Third, we reviewed every identified article to determine if the dependent variable and focus of the study was reducing problem behavior. Multi-study articles that included at least one study with problem behavior as a dependent variable were included. Fourth, we included only studies that applied BMT or RaC prospectively. We defined prospective applications as (1) collecting baseline data (e.g., rate of problem behavior, number of reinforcers delivered) and (2) using the collected baseline data in an equation in order to empirically derive portions of treatment (e.g., dosage of treatment sessions, schedule thinning values) or make predictions before conducting any treatment sessions. We also included studies that collected both *baseline and treatment* data, and then refined treatment based on calculations in a model. Three experimenters completed the initial search. A second, independent rater (a BCBA-D) independently screened 25 randomly selected articles from the full pool, representing 35% of the total identified articles. Agreement on inclusion decisions was 100%, indicating strong reliability of the initial screening process.

## 3. Results and Discussion

**Figure 1:**
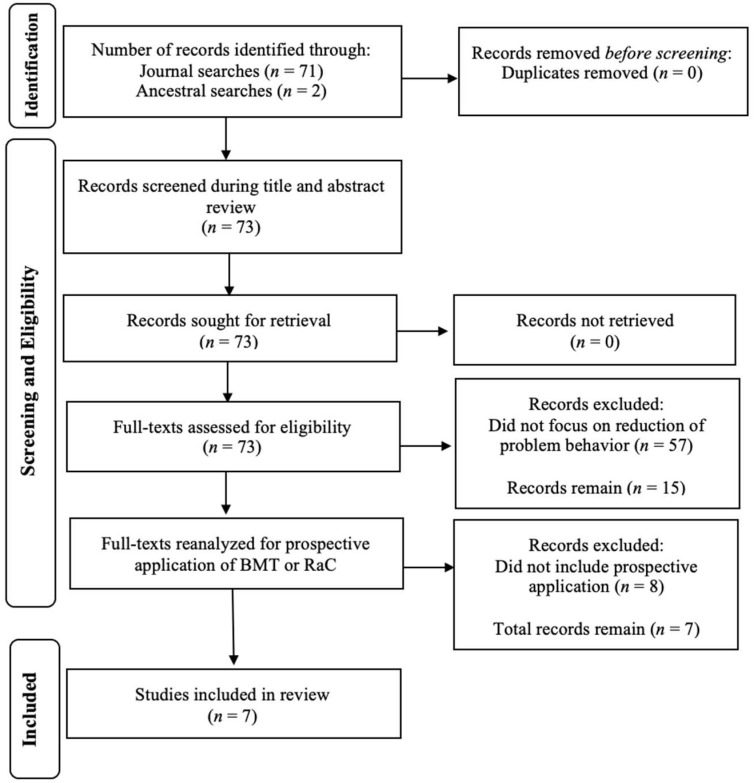
Review flowchart. Note: BMT = Behavioral Momentum Theory, RaC = Resurgence as Choice. Search procedures and flowcharts are commensurate with the PRISMA extension for scoping reviews (PRISMA-ScR; Tricco et al., 2018).

We identified 70 articles that mentioned BMT or RaC in either the title, keywords, or abstract published between 2014 and 2024. We then identified two additional relevant articles via the ancestral search^2^. Thus, 72 articles were initially included. Of those 72 articles, 15 (20.8%) focused on reduction in problem behavior. Of those 15, 7 (46.6%) included a prospective application of BMT or RaC to predict treatment effectiveness or make treatment refinements. Those seven articles were: Pritchard et al. (2014), Fisher et al. (2018, 2019), Greer et al. (2020, 2023, 2024), and Irwin Helvey et al. (2023). Figure 1 depicts our search flowchart based on PRISMA-ScR guidelines (Tricco et al., 2018).

In three of the seven articles that included a prospective application, the treatments designed based on the prospective application of these models were more effective and durable compared to treatments that were not designed based on the models (i.e., Fisher et al., 2018, 2019; Pritchard et al., 2014). Two of the seven articles evaluated the extent to which different durations of treatment exposure (or rather, exposure to extinction) impacted resurgence (i.e., Greer et al., 2020, 2023). The findings from both Greer et al. (2020) and Greer et al. (2023) showed minimal differences in resurgence between two manipulations of treatment duration based on BMT. Thus, the researchers were not explicitly testing if a treatment based on BMT was better than a treatment not based on BMT, but rather two different treatments both based on BMT. Similarly, Irwin Helvey et al. (2023) evaluated different rates of alternative reinforcement informed by BMT and found no reliable differences in resurgence. Most recently, Greer et al. (2024) evaluated the extent to which the size of the decrease in alternative reinforcement would impact resurgence. In general, the results of their experiments aligned well with the predictions of RaC. When a fixed progression of decreases in alternative reinforcement occurred, participants showed low levels of problem behavior (Experiment 1). In contrast, participants who experienced larger decreases in alternative reinforcement earlier in their treatments engaged in higher levels of resurgence (Experiment 2).

To our knowledge, this is the first review in which researchers quantified the extent to which BMT and RaC are being applied prospectively within the assessment and treatment of problem behavior. Our results provide an initial benchmark to which future results can be compared. Basic and translational research focused on enhancing treatment durability and further understanding and preventing relapse indicates that treatments can be improved by using quantitative models like BMT or RaC (Craig et al., 2019; Greer et al., 2020; Greer & Shahan, 2019; Nevin et al., 2017; Shahan et al., 2020; Shahan & Greer, 2021). That is, rather than using quantitative models to describe environment–behavior relations retrospectively, these models may be used to develop treatments before their implementation. This notion is supported by recent, albeit sparse yet growing, applied research in which the models were used and applied in a prospective fashion resulting in more durable treatments (e.g., Fisher et al., 2018, 2019). The use of these models in the treatment of problem behavior is also valued by the scientific community at large, as evidenced by federal funding for projects on the topic (Fisher, 2015–2020). Unfortunately, based on our review, these models are very rarely applied prospectively despite their apparent enormous benefits (i.e., improving the durability of treatments). Indeed, this seems to be evident in the sheer number of articles (i.e., 72 total) that we identified that mentioned the models in some capacity, but either (a) did not focus on problem behavior or (b) failed to incorporate a prospective application.

Research-to-practice gaps are very common in applied fields, including applied behavior analysis (e.g., Mace & Critchfield, 2010). Successful translation is important because knowing and understanding basic principles of behavior allows us to anticipate treatment effects more precisely and enhance clinical outcomes (Critchfield & Reed, 2009). Such developments provide applied researchers and clinicians with new frameworks for understanding (and preventing) the relapse of problem behavior.

Many variables affect the successful translation of quantitative models to applied practice and implementation (see Critchfield & Reed, 2009). A number of these are likely relevant to this discussion of relapse and the prospective application of BMT and RaC. Broadly, there are two different barriers that may make it difficult to bring these models to bear in applied contexts. First, many of the procedural arrangements used to study relapse phenomena in basic settings are difficult to arrange in the real world. That is, many of the core features of behavioral persistence preparations commonly used in laboratory settings (e.g., use of concurrent VI-VI schedules, changeover delays, discrete response alternatives, easily quantifiable reinforcers) can be difficult to arrange in naturalistic contexts or applied situations (e.g., automatically maintained behavior). Some of these issues have previously been articulated with respect to other quantitative models (e.g., Critchfield & Reed, 2009), so we will not relitigate these points here. A second possible barrier may be that the practical significance of the parameters used in BMT and RaC (i.e., *A*, l, *x*) might not be immediately obvious or clear. Many quantitative models contain variables or fitted parameters that can readily be explained and understood by reference to the equations but are hard to understand in a practical sense. In other words, a limitation of some quantitative models is that their fitted parameters do not neatly map onto everyday circumstances and clinical situations—these issues are not unique to only BMT and RaC (Critchfield & Reed, 2009). Below, we briefly outline a few additional barriers relevant to the application of these models along with some potential solutions.

### 3.1. Potential Barrier: Lack of Familiarity with Quantitative Models

BMT and RaC are technically sophisticated; their understanding and use require at least a cursory handling of the equations they entail (Critchfield & Reed, 2009). However, skillsets to proficiently use them may not be in the repertoire of many behavior analysts working in applied settings. Rather, basic and translational researchers are the individuals who currently seem best equipped to employ these models in a prospective manner (Nevin, 2008). This would appear especially true given that explicit training in quantitative models, such as BMT and RaC, is not required to work in applied settings with individuals that display problem behavior specifically (Behavior Analyst Certification Board [BACB], 2022), as well as to obtain graduate degrees in behavior analysis broadly (Association for Behavior Analysis International [ABAI], n.d.).

#### 3.1.1. Potential Solution: Develop and Disseminate Model-Specific Training and Tutorials

Given the complexity of quantitative models, if one is to expect them to be used widely in the treatment of problem behavior, one must also make those models and requisite skills easily accessible (Critchfield & Reed, 2009). One way to improve the use of and skillsets related to models is through the provision of tutorials—either via publications or conference presentations (Greer, 2021; Greer et al., 2016a; Laureano & Falligant, 2023). This approach is supported by examples of other quantitative models (e.g., Reed et al., 2013) and related research in the area of quantitative data analysis methods in which individuals provide tutorials with explicit instructions for using advanced data-analytic procedures (e.g., Turgeon & Lanovaz, 2020). Thus, providing workshops on tutorials, guides, and aides for the use of quantitative models is a logical step to increasing their prevalence. Such materials might best serve researchers and clinicians by being either illustrative or providing templates with which individuals can enter various parameters into the model. Further, this could open a wide area for researchers; namely, what are the best methods for training individuals to use quantitative models in a prospective manner?

#### 3.1.2. Potential Solution: Expanding Training in Quantitative Models Through Coursework and Certification Standards

Although some programs provide formal training in quantitative models of behavior (e.g., ABSC 936: *Quantitative Analysis of Behavior* at the University of Kansas), such opportunities remain limited (Association for Behavior Analysis International [ABAI], n.d.). Furthermore, training in quantitative models is not currently required under the BACB Test Content Outline (Behavior Analyst Certification Board [BACB], 2022), leaving a significant gap in standardized education for behavior analysts. To address this, educators—particularly those teaching in ABAI-accredited programs or Verified Course Sequences—should consider developing coursework specifically focused on quantitative modeling and behavioral analysis. Additionally, future iterations of the BACB Test Content Outline could incorporate quantitative models to ensure that foundational training in these methods becomes a standard component of professional preparation.

One challenge in applying these models is the need to collect data to directly inform—or otherwise estimate—parameter values to generate meaningful predictions. This requires either specific assessments or data sources that can provide reliable estimates of key parameters (e.g., sensitivity, bias, specific activation) or making empirically and theoretically sound approximations based on available data, clinical observations, or other contextual factors. This complexity underscores the fact that more training alone may not entirely resolve barriers to their application. However, familiarity with these models and their underlying parameters is a necessary first step. Understanding how these models function and what their parameters represent provides a critical foundation for making informed approximations and guiding data collection strategies to refine predictions.

Learning to use quantitative models represents an important behavioral cusp, granting behavior analysts access to a powerful analytical framework that extends beyond Resurgence as Choice (RaC) and Behavioral Momentum Theory (BMT). These models can be applied to optimize reinforcer selection (e.g., Mathematical Principles of Reinforcement; Killeen & Sitomer, 2003), structure reinforcement-based interventions without extinction (e.g., concatenated matching law; Baum & Rachlin, 1969), improve understanding of extinction-induced behavior change (e.g., temporally weighted matching law; Shahan, 2022), and enhance instrumental behavior using activity-based reinforcers (e.g., disequilibrium theory; Dowdy & Jacobs, 2019). Expanding training in quantitative modeling would strengthen the analytical skillsets of behavior analysts, fostering the integration of data-driven decision-making into clinical and applied practice.

## 4. Concluding Remarks

A necessary element of basic and translational research regarding quantitative models involves further refinement of existing models and the development of new ones. That is, as new evidence emerges, existing quantitative models with bodies of support may be discarded for more updated and comprehensive models^3^. The continued evolution and refinement of these models is necessary. However, it may be difficult for clinicians and other applied researchers to track each new development. Thus, there is an enormous opportunity to help close the research-to-practice gap by disseminating these advances in applied outlets and further translating BMT and RaC in a consumable manner to those working in applied settings. One suggestion for closing this gap could be special issues on quantitative models and their application to treatment in journals such as *BAP* or *JABA*, which are likely to capture a more applied audience compared to *JEAB* or other more basic journals. Further, reviewers and associate editors should work with authors submitting quantitative submissions to give them the broadest reach possible.

There is evidence that prospectively applying quantitative models will result in more durable treatments compared to those not based on models (Fisher et al., 2018). Based on our review, BMT and RaC are very rarely applied prospectively as they relate to problem behavior. This may be due to some of the barriers mentioned above. However, it is important to note two alternative hypotheses (and limitations). First, our review included articles published within the past 10 years and from 10 journals that published studies related to *both* problem behavior and BMT or RaC. It is possible prospective applications were published prior to 2014 and in other journals, though this seems unlikely. Nevertheless, future researchers might consider conducting a systematic literature review that does not truncate the years of publication and initiates a database search. Second, it could be the case that clinicians are prospectively applying BMT and RaC in practice and choosing not to publish those cases—this too appears unlikely. However, if clinicians are in fact using these models prospectively in practice, we strongly encourage them to submit those cases and results for publication, as, from our perspective, those would be nice contributions to the literature.

There is another point relevant to the adoption and prospective application of BMT and RaC specifically, and quantitative models broadly, in the treatment of problem behavior that warrants discussion. Like all sciences, behavior analysis is ever-evolving, with new discoveries, empirical evidence, and conceptual perspectives emerging on a regular basis. For example, BMT has a long and enduring history as a theoretical and conceptual model of behavior. However, it has undergone recent scrutiny, and contemporary studies have yielded important insights regarding the limitations of the model (see (Craig, 2023) for a thorough discussion). Moreover, although it is a relatively new model, RaC has continued to evolve in a quick manner in a short amount of time (Shahan & Craig, 2017; Shahan et al., 2020). Importantly, the emerging RaC-related research continues to be promising and supportive, meaning RaC and its related family of models (e.g., temporally weighted matching law) are likely to be an enduring quantitative model (e.g., Fisher et al., 2023; Shahan, 2022; Shahan et al., 2024). Regardless, our point here related to the adoption of quantitative models in applied practice is that basic and translational researchers are likely to conduct studies in a manner that outpaces clinicians’ adoption and use of contemporary models in applied practice. Perhaps it is, therefore, unsurprising that quantitative models are rarely used in applied practice—it is reasonable to question how or why a given model should be adopted if a newer, more advanced model is soon to follow.

The issue of prospective application seems not to be unique to BMT and RaC; however, future research in this area may be warranted. Regardless, it seems that, at present, BMT and RaC are more applicable as a conceptual framework for refining treatments rather than as an empirical method (cf. Critchfield & Reed, 2009; see Schieltz et al., 2017; Wacker et al., 2011 for examples). We hope the discussion contained herein helps to bridge the research-to-practice gap regarding quantitative models and bring more awareness to clinicians surrounding their utility, as well as to basic and translational researchers surrounding some issues related to bridging the aforementioned gap.

## Data Availability

All analyses were conducted on publicly available data. Additional data beyond those described herein are available from the corresponding author upon reasonable request.

## Notes

1 This also includes if BMT or RaC was mentioned in part. For example, if an article had the keyword “behavioral momentum”, those were included in the first step. For the purpose of this review, we did not make a distinction between RaC (Shahan & Craig, 2017), and Resurgence as Choice in Context (RaC2; Shahan et al., 2020).

2 The articles included through the ancestral search (a) included, “…A prospective analysis”, in its title (i.e., Greer et al., 2024) or (b) otherwise met every other criteria for inclusion sans mentioning a model in the keyword, title, or abstract. Therefore, we reviewed those articles. Although BMT or RaC was not mentioned in the title, keywords, or abstract, those two articles met every other inclusion criteria. Given the scant nature of this research, it seemed wise to include them.

3 Consider BMT, RaC, and Resurgence as Choice in Context (RaC2). BMT has substantial empirical evidence; it has led to further understanding of behavioral persistence and relapse and is one of the models that has been successfully applied in a prospective manner. However, researchers have recently identified several shortcomings of BMT (e.g., failure to fully account for disruptive effects of alternative reinforcement; see (Shahan & Craig, 2017) and (Shahan & Sweeney, 2011) for discussions). Thus, RaC was developed to account for the shortcomings of BMT. Shortly thereafter, RaC2 emerged to better account for the local effects of rapid alternation between different schedules of alternative reinforcement and other procedural arrangements.

## Abbreviations

The following abbreviations are used in this manuscript:

RaC	Resurgence as Choice
BMT	Behavioral Momentum Theory
JABA	Journal of Applied Behavior Analysis
JEAB	Journal of the Experimental Analysis of Behavior

## References

[B1-behavsci-15-00688] Association for Behavior Analysis International [ABAI] (n.d.). Verified course sequence program.

[B2-behavsci-15-00688] Baum W. M., Rachlin H. C. (1969). Choice as time allocation. Journal of the Experimental Analysis of Behavior.

[B3-behavsci-15-00688] Behavior Analyst Certification Board [BACB] (2022). BCBA test content outline.

[B4-behavsci-15-00688] Briggs A. M., Greer B. D., Maragakis A., Drossel C., Waltz T. J. (2021). Intensive behavioral intervention units. Applications of behavior analysis to healthcare and beyond.

[B5-behavsci-15-00688] Craig A. R. (2023). Resistance to change, of behavior and of theory. Journal of the Experimental Analysis of Behavior.

[B6-behavsci-15-00688] Craig A. R., Sullivan W. E., Roane H. S. (2019). Further evaluation of a nonsequential approach to studying operant renewal. Journal of the Experimental Analysis of Behavior.

[B7-behavsci-15-00688] Critchfield T. S., Reed D. D. (2009). What are we doing when we translate from quantitative models. The Behavior Analyst.

[B8-behavsci-15-00688] Curiel E. S. L., Kranak M. P., Fielding C., Curiel H., Miller M. M. (2023). Behavior analysis in college classrooms: A scoping review. Behavioral Interventions.

[B9-behavsci-15-00688] Dowdy A., Jacobs K. W. (2019). An empirical evaluation of the disequilibrium model to increase independent seatwork for an individual diagnosed with autism. Behavior Analysis in Practice.

[B10-behavsci-15-00688] Falligant J. M., Kranak M. P., McNulty M. K., Schmidt J. D., Hausman N. L., Rooker G. W. (2021). Prevalence of renewal of problem behavior: Replication and extension to an inpatient setting. Journal of Applied Behavior Analysis.

[B11-behavsci-15-00688] Fisher W. W. (2015–2020). Preventing relapse of destructive behavior using behavioral momentum theory. *Project No. 7R01HD083214-06 [Grant]*.

[B12-behavsci-15-00688] Fisher W. W., Greer B. D., Fuhrman A., Saini V., Simmons C. (2018). Minimizing resurgence of destructive behavior using behavioral momentum theory. Journal of Applied Behavior Analysis.

[B13-behavsci-15-00688] Fisher W. W., Greer B. D., Mitteer D. R., Fuhrman A. M. (2022). Translating quantitative theories of behavior into improved clinical treatments for problem behavior. Behavioural Processes.

[B14-behavsci-15-00688] Fisher W. W., Greer B. D., Shahan T. A., Norris H. M. (2023). Basic and applied research on extinction bursts. Journal of Applied Behavior Analysis.

[B15-behavsci-15-00688] Fisher W. W., Saini V., Greer B., Sullivan W., Roane H., Fuhrman A., Craig A., Kimball R. (2019). Baseline reinforcement rate and resurgence of destructive behavior. Journal of the Experimental Analysis of Behavior.

[B16-behavsci-15-00688] Greer B. D. (2021). SQAB tutorial: Using quantitative theories of relapse to improve functional communication. Association for Behavior Analysis International 47th Annual Convention.

[B17-behavsci-15-00688] Greer B. D., Fisher W. W., Retzlaff B. J., Fuhrman A. M. (2020). A preliminary evaluation of treatment duration on the resurgence of destructive behavior. Journal of the Experimental Analysis of Behavior.

[B18-behavsci-15-00688] Greer B. D., Fisher W. W., Romani P. W., Saini V. (2016a). Behavioral momentum theory: A tutorial on response persistence. The Behavior Analyst.

[B19-behavsci-15-00688] Greer B. D., Fisher W. W., Saini V., Owen T. M., Jones J. K. (2016b). Functional communication training during reinforcement schedule thinning: An analysis of 25 applications. Journal of Applied Behavior Analysis.

[B20-behavsci-15-00688] Greer B. D., Shahan T. A. (2019). Resurgence as choice: Implications for promoting durable behavior change. Journal of Applied Behavior Analysis.

[B21-behavsci-15-00688] Greer B. D., Shahan T. A., Fisher W. W., Mitteer D. R., Fuhrman A. M. (2023). Further evaluation of treatment duration on the resurgence of destructive behavior. Journal of Applied Behavior Analysis.

[B22-behavsci-15-00688] Greer B. D., Shahan T. A., Irwin Helvey C., Fisher W. W., Mitteer D. R., Fuhrman A. M. (2024). Resurgence of destructive behavior following decreases in alternative reinforcement: A prospective analysis. Journal of Applied Behavior Analysis.

[B23-behavsci-15-00688] Hustyi K. M., Ryan A. H., Hall S. S. (2023). A scoping review of behavioral interventions for promoting social gaze in individuals with autism spectrum disorder and other developmental disabilities. Research in Autism Spectrum Disorders.

[B24-behavsci-15-00688] Irwin Helvey C., Fisher W. W., Greer B. D., Fuhrman A. M., Mitteer D. R. (2023). Resurgence of destructive behavior following differential rates of alternative reinforcement. Journal of Applied Behavior Analysis.

[B25-behavsci-15-00688] Kaur J., Kranak M. P., Mitteer D. R., Melanson I., Fahmie T. A. (2025). A scoping review of consecutive controlled case series studies. Journal of Applied Behavior Analysis.

[B26-behavsci-15-00688] Killeen P. R., Sitomer M. T. (2003). MPR. Behavioural Processes.

[B27-behavsci-15-00688] Kranak M. P., Falligant J. M. (2021). Further investigation of resurgence following schedule thinning: Extension to an inpatient setting. Behavioral Interventions.

[B28-behavsci-15-00688] Laureano B., Falligant J. M. (2023). Modelling behavioral persistence with resurgence as choice in context (RaC^2^): A tutorial. Behavior Analysis in Practice.

[B29-behavsci-15-00688] Mace F. C., Critchfield T. S. (2010). Translational research in behavior analysis: Historical traditions and imperative for the future. Journal of the Experimental Analysis of Behavior.

[B30-behavsci-15-00688] Mitteer D. R., Greer B. D., Randall K. R., Haney S. D. (2022). On the scope and characteristics of treatment relapse when treating destructive behavior. Journal of Applied Behavior Analysis.

[B31-behavsci-15-00688] Muething C., Call N., Pavlov A., Ringdahl J., Gillespie S., Clark S., Mevers J. L. (2020). Prevalence of renewal of problem behavior during context changes. Journal of Applied Behavior Analysis.

[B32-behavsci-15-00688] Munn Z., Peters M. D. J., Stern C., Tufanaru C., McArthur A., Aromataris E. (2018). Systematic review or scoping review?: Guidance for authors when choosing between a systematic and a scoping review approach. BMC Medical Research Methodology.

[B33-behavsci-15-00688] Nevin J. A. (2002). Measuring behavior momentum. Behavioural Processes.

[B34-behavsci-15-00688] Nevin J. A. (2008). Control, prediction, order, and the joys of research. Journal of the Experimental Analysis of Behavior.

[B35-behavsci-15-00688] Nevin J. A., Craig A. C., Cunningham P. J., Podlesnik C. A., Shahan T. A., Sweeney M. M. (2017). Quantitative models of persistence and relapse from the perspective of behavioral momentum theory: Fits and misfits. Behavioural Processes.

[B36-behavsci-15-00688] Nevin J. A., Grace R. C. (2000). Behavioral momentum and the law of effect. Behavioral and Brain Sciences.

[B37-behavsci-15-00688] Nevin J. A., Shahan T. A. (2011). Behavioral momentum theory: Equations and applications. Journal of Applied Behavior Analysis.

[B38-behavsci-15-00688] Pritchard D., Hoerger M., Mace F. C. (2014). Treatment relapse and behavior momentum theory. Journal of Applied Behavior Analysis.

[B39-behavsci-15-00688] Reed D. D., Niileksela C. R., Kaplan B. A. (2013). Behavioral economics: A tutorial for behavior analysts in practice. Behavior Analysis in Practice.

[B40-behavsci-15-00688] Saini V., Fisher W. W., Pisman M. D. (2017). Persistence during and resurgence following noncontingent reinforcement implemented with and without extinction. Journal of Applied Behavior Analysis.

[B41-behavsci-15-00688] Schieltz K. M., Wacker D. P., Ringdahl J. E., Berg W. K. (2017). Basing assessment and treatment of problem behavior on behavioral momentum theory: Analyses of behavioral persistence. Behavioural Processes.

[B42-behavsci-15-00688] Shahan T. A. (2022). A theory of the extinction burst. Perspectives on Behavior Science.

[B43-behavsci-15-00688] Shahan T. A., Browning K. O., Nall R. W. (2020). Resurgence as choice in context: Treatment duration and on/off alternative reinforcement. Journal of the Experimental Analysis of Behavior.

[B44-behavsci-15-00688] Shahan T. A., Craig A. R. (2017). Resurgence as choice. Behavioural Processes.

[B45-behavsci-15-00688] Shahan T. A., Greer B. D. (2021). Destructive behavior increases as a function of reductions in alternative reinforcement during schedule thinning: A retrospective qualitative analysis. Journal of the Experimental Analysis of Behavior.

[B46-behavsci-15-00688] Shahan T. A., Sutton G. M., Allsburg J. V., Avellaneda M., Greer B. D. (2024). Resurgence following higher or lower quality alternative reinforcement. Journal of the Experimental Analysis of Behavior.

[B47-behavsci-15-00688] Shahan T. A., Sweeney M. M. (2011). A model of resurgence based on behavioral momentum theory. Journal of the Experimental Analysis of Behavior.

[B48-behavsci-15-00688] Smith S. W., Greer B. D., Matson J. L. (2023). Behavioral momentum theory. Handbook of applied behavior analysis: Integrating research into practice.

[B49-behavsci-15-00688] Tricco A. C., Lillie E., Zarin W., O’Brien K. K., Colquhoun H., Levac D., Moher D., Peters M. D. J., Horsley T., Weeks L., Hempel S., Akl E. A., Chang C., McGowan J., Stewart L., Hartling L., Aldcroft A., Wilson M. G., Garritty C., Straus S. E. (2018). PRISMA extension for scoping reviews (PRISMA-ScR): Checklist and explanation. Annals of Internal Medicine.

[B50-behavsci-15-00688] Trump C. E., Herrod J. L., Ayers K. M., Ringdahl J. E., Best L. (2021). Behavior momentum theory and humans: A review of the literature. The Psychological Record.

[B51-behavsci-15-00688] Turgeon S., Lanovaz M. J. (2020). Tutorial: Applying machine learning in behavioral research. Perspectives on Behavior Science.

[B52-behavsci-15-00688] Wacker D. P., Harding J. W., Berg W. K., Lee J. F., Schieltz K. M., Padilla Y. C., Nevin J. A., Shahan T. A. (2011). An evaluation of persistence of treatment effects during long-term treatment of destructive behavior. Journal of the Experimental Analysis of Behavior.

